# A community-based cluster randomized survey of noncommunicable disease and risk factors in a peri-urban shantytown in Lima, Peru

**DOI:** 10.1186/1472-698X-14-19

**Published:** 2014-05-21

**Authors:** Kristen Heitzinger, Silvia M Montano, Stephen E Hawes, Jorge O Alarcón, Joseph R Zunt

**Affiliations:** 1 Department of Epidemiology, University of Washington, Seattle, WA, USA; 2 U.S. Naval Medical Research Unit No. 6, Callao, Peru; 3 Instituto de Medicina Tropical “Daniel A. Carrión”, Universidad Nacional Mayor de San Marcos, Lima, Peru; 4 Departments of Neurology, Global Health, and Medicine, University of Washington, Seattle, WA, USA

**Keywords:** Peru, Slum, Shantytown, Noncommunicable disease

## Abstract

**Background:**

An estimated 863 million people–a third of the world’s urban population–live in slums, yet there is little information on the disease burden in these settings, particularly regarding chronic preventable diseases.

**Methods:**

From March to May 2012, we conducted a cluster randomized survey to estimate the prevalence of noncommunicable diseases (NCDs) and associated risk factors in a peri-urban shantytown north of Lima, Peru. Field workers administered a questionnaire that included items from the WHO World Health Survey and the WHO STEPS survey of chronic disease risk factors. We used logistic regression to assess the associations of NCDs and related risk factors with age and gender. We accounted for sampling weights and the clustered sampling design using statistical survey methods.

**Results:**

A total of 142 adults were surveyed and had a weighted mean age of 36 years (range 18–81). The most prevalent diseases were depression (12%) and chronic respiratory disease (8%), while lifetime prevalence of cancer, arthritis, myocardial infarction, and diabetes were all less than 5%. Fifteen percent of respondents were hypertensive and the majority (67%) was unaware of their condition. Being overweight or obese was common for both genders (53%), but abdominal obesity was more prevalent in women (54% vs. 10% in men, p < 0.001). Thirty-five percent of men binge drank and 34% reported current smoking; these behaviors were less common among women (4% binge drank, p < 0.001; 8% smoked, p = 0.002). Increasing age was associated with an increased risk of abdominal obesity (Odds Ratio (OR) = 1.04, 95% CI = 1.01, 1.07, p = 0.02), hypertension (OR = 1.06, 95% CI = 1.02, 1.10, p = 0.006), arthritis (OR = 1.07, 95% CI = 1.03, 1.11, p < 0.001) and cancer (OR = 1.13, 95% CI = 1.07, 1.20, p < 0.001) in adjusted models. The prevalences of other NCDs and related risk factors were similar when stratified by age or gender.

**Conclusions:**

This study underlines the important burden of noncommunicable disease in informal settlements in Peru and suggests that prevention and treatment interventions could be optimized according to age and gender.

## Background

In developing countries, an estimated 863 million people live in slums, areas characterized by poor quality or informal housing, unhealthy living conditions, poverty, and marginalization from the formal health sector [1]. Because slum dwellers represent a third of the urban population of low- and middle-income countries, addressing intra-urban health inequities is an important challenge facing cities in the developing world [1,2]. Little is known about the burden of disease in slums and low-income informal settlements because, due to their unofficial status and lack of resources, they are more frequently excluded from governmental epidemiologic surveillance and research studies. Additionally, health inequalities between slums and adjacent urban areas may be masked by their inclusion in a single surveillance catchment area, which has led to repeated calls for more robust intra-urban morbidity and mortality data [2-6]. Drawing on social epidemiologic theory, a high prevalence of a range of noncommunicable diseases (cardiovascular and chronic respiratory disease, diabetes, mental illness, arthritis, and cancer) and related risk factors (overweight and obesity, poor nutrition, harmful drinking, smoking, and hypertension) is expected in a slum population due to the particularly adverse factors present within each component of the WHO conceptual framework of social determinants of health [7]. This framework conceptualizes disease risk and risk behaviors as being influenced by three major components: the socioeconomic-political context, the structural determinants of socioeconomic position, and the intermediary determinants of health [7]. Slums are characterized by urbanization, a lack of urban planning, overcrowding, and exclusion from social, health, and other services; such a socioeconomic-political context could be expected to have a higher prevalence of noncommunicable diseases due to the increased exposure to modifiable risk factors such as increased intake of fat and sugar associated with an urban lifestyle, few opportunities for physical activity due to limited space and insecurity, and a high prevalence of undiagnosed chronic conditions due to a lack of access to primary care services and health education. Slum dwellers also typically occupy a low socioeconomic position, and poverty and lower education levels are strongly linked to the development of NCDs [8]. Primary intermediary factors that are expected to be prevalent in a slum setting and contribute to the development of noncommunicable disease include inadequate access to sanitation and other infrastructure [5], insecure residential status [5], and exposure to violence and crime. In informal settlements, chronic noncommunicable diseases are at particular risk of going undetected by formal health registries until presentation in a late stage of disease or death; this has been attributed to a lack of access to health services and inadequate or inappropriate care when services are sought [4,5]. This pattern of health-seeking behavior typically results in an undue human cost and financial burden on existing health systems [3,4], underscoring the need for noncommunicable disease (NCD) data to advise health interventions targeting the urban poor.

In Peru, a middle-income country with a growing burden of NCD [9,10], approximately forty percent of the population of the capital city, Lima, lives in low-income informal settlements. Limited research has investigated the epidemiology of NCD in populations living in informal settlements in Lima. The PERU-MIGRANT study was conducted in rural-to-urban migrants and lifelong urban residents living in a shantytown south of Lima [11]; although not designed to yield population-based disease estimates, data from the shantytown segment of the PERU-MIGRANT cohort provided evidence of a significant risk of cardiovascular disease. The prevalence of overweight or obesity, hypertension, and diabetes were estimated to be 67-71%, 13-30%, and 2-5%, respectively [12] and these results were similar to those of obesity and hypertension studies in other populations in peri-urban Lima [13,14]. A probable mood disorder (defined by a validated questionnaire) was identified in approximately a third (33-38%) of the PERU-MIGRANT shantytown cohort, suggesting an important burden of mental illness in these informal settlements [15]. Although noncommunicable chronic respiratory disease has not been studied in an adult Peruvian shantytown population, research in adolescents indicates that this may additionally contribute to the NCD burden among adults living in these communities [16]. Prior studies in informal settlements in Peru have identified gender and age as determinants of NCD and related risk behaviors. The prevalences of binge drinking and smoking were significantly higher among men [17,18], while women were more likely to be obese (assessed by either body mass index or waist circumference) and have metabolic syndrome [13,19,20]. Increasing age was associated with hypertension in an adult shantytown population [13]. As a part of a population-based health needs evaluation in a Peruvian shantytown, we aimed to measure the prevalence of NCD and related risk factors and to evaluate their associations with age and gender in order to inform the design of future interventions in this community.

## Methods

### Study setting

The study was conducted from March to May 2012 in Lomas de Zapallal, a shantytown located in a peri-urban area north of Lima, Peru. Lomas de Zapallal began to be settled approximately 20 years ago and has an estimated population of 30,000, although it continues to expand geographically into the uninhabited surrounding hills. A public water and sanitation system was installed in the community in 2009 and currently services about 90% of the population, but there is an important lack of other public services and access to health care. A research study in schoolchildren was previously conducted in this community [21], and a collaborative effort led by the Universidad Nacional Mayor de San Marcos, the University of Washington, and Architects Without Borders-Seattle to design and construct a healthier learning environment at a community school is ongoing [22].

### Study design and procedures

Study subjects were sampled using a two-step cluster sampling method. A building density map of Lomas de Zapallal was used to divide the community into 30 clusters of approximately equal population size, and households were randomly selected from each cluster by identifying a random location within the cluster, interviewing the eligible household nearest to that location, and proceeding to interview adjacent eligible households until meeting the required number of households per cluster, according to the World Health Organization EPI Methodology [23]. We calculated a sample size of 135 households was needed to yield prevalence estimates with a minimum precision of ±12.5%, assuming a design effect of 2.2 and given that, in contrast to the EPI Method, all clusters were sampled as compared to a random sample [24]. We therefore aimed to sample 4 or 5 households from each cluster in order to ensure a representative sample and adequate sample size. Adults aged 18 and older who spoke Spanish and who were able to comply with study procedures were eligible to participate and an adult household member was chosen from each household for study participation using a random number table. Three attempts were made to survey houses in which the selected respondent was initially unavailable. Houses were most frequently surveyed on Sundays in an effort to maximize the probability of contacting selected potential participants.

Informed consent was obtained from each subject prior to the start of any study procedures. Field workers administered a comprehensive health questionnaire regarding health history, behaviors, and sociodemographic information (Additional file 1). This questionnaire included selected questions from the World Health Survey and all of the core questions of the WHO STEPS survey on noncommunicable disease risk factors [25] with the exception of the questions regarding physical activity and biochemical measures, which were excluded to decrease the length of the interview. This questionnaire was pilot tested in a small group of community members having similar sociodemographic characteristics as the study population.

Anthropometric measures including height, weight, blood pressure, and waist circumference were collected for each subject. Participants were requested to remove their shoes prior to measurement of height and weight and were weighed wearing light clothing. To improve accuracy, blood pressure was measured with the subject seated and the mean of two measures (taken at least 3 minutes apart) was used in analyses. Waist circumference was not measured in women who reported currently being pregnant. Ethical approval for this study was obtained from the Institutional Review Board of the Universidad Nacional Mayor de San Marcos. The Institutional Review Board of the University of Washington determined that this study did not meet the federal definition of research and it was therefore exempt from review.

### Measures

NCD conditions were defined as follows:

1) Depression, chronic respiratory disease, arthritis, cancer, and myocardial infarction: The presence of one of these conditions was defined as an affirmative answer to the question “have you ever been told by a doctor or other health professional that you have or had depression, chronic respiratory disease, arthritis, cancer or myocardial infarction?”

2) Diabetes: Self-reported diabetes (an affirmative answer to the question “have you ever been told by a doctor or other health professional that you have diabetes?”) and displayed diabetes medications to the field worker for documentation.

Physiological risk factors for NCD were defined as follows:

1) Obesity: We used two measures to define obesity: body mass index (BMI) and abdominal obesity. Per World Health Organization (WHO) guidelines, BMI was categorized as normal weight (18.5 ≤ BMI ≥ 24.9), overweight (25.0 ≤ BMI ≥ 29.9) or obese (BMI ≥ 30.0) [26]. Abdominal obesity was measured to complement the definition of obesity using BMI because, although BMI is a widely used measure of obesity and has a well established association with cardiovascular disease (CVD), diabetes, and cancer risk, there is evidence that abdominal obesity better predicts CVD risk [27]. Per the WHO definition, waist circumference of >102 cm in men and >88 cm in women was defined as having abdominal obesity [27]. In a 2010 study in Peru, cutoffs of >97 cm in men and >87 cm in women were optimal to detect subclinical and manifest cardiovascular disease [28]; we therefore included both definitions in our analyses.

2) Hypertension: Participants were considered as having hypertension if the mean systolic blood pressure measure was ≥140 mmHg and/or the mean diastolic blood pressure measure was ≥90 mmHg. Participants were also considered as having hypertension if they reported a physician diagnosis and could present hypertensive medications to the field worker for documentation.

Behavioral risk factors for NCD were defined as follows:

1) Tobacco use: Participants were questioned about current smoking (yes/no) and frequency of smoking (daily/less frequent). Daily smokers were questioned about frequency of use of specific tobacco products and about smoking history.

2) Harmful alcohol consumption: Participants were questioned about lifetime alcohol use, frequency of alcohol use within the previous 12 months, and frequency of alcohol use within the past 30 days. Harmful alcohol consumption (binge drinking) was defined for women as having consumed 4 or more standard alcoholic drinks on a single occasion in the last 30 days, and for men, as having consumed 5 or more drinks. Pictures of examples of standard alcoholic drinks were shown to participants to clarify quantities of alcohol.

3) Inadequate fruit and vegetable consumption: Participants were asked to estimate the number of days they consumed fruit and the average number of portions of fruit consumed on one of those days. Similar questions were asked regarding vegetable consumption. The average daily number of portions of fruits and vegetables consumed was calculated from these variables. Daily fruit and vegetable consumption of less than 5 portions was considered to be inadequate, per the WHO recommendations of NCD behavioral risk factor indicators [8].

### Statistical analysis

We used statistical survey methods (the *svy* command in STATA 13.1) to account for survey weights and the clustered sampling design. Because age and sex were collected for all adults in contacted households including those that did not participate, the age and sex distribution of the participants was compared to those of the non-participants and the data were weighted back to the population sex distribution (49.9% male, 50.1% female) to correct for the non-response of males selected to participate. Descriptive statistics were generated using cross-tabulations and Chi-square tests were used to assess significant differences between groups. Logistic regression was used to determine whether participant age and sex were associated with the odds of chronic disease diagnosis or chronic disease risk behaviors. Odds ratios and 95% confidence intervals (95% CIs) were estimated for these associations. Sex and (continuous) age were included as a priori confounders in adjusted models of the associations of sex and age, respectively. Variables known to be associated with selected NCD and risk factors were also considered as potential confounders in adjusted models; these variables included educational attainment (none, completed elementary school, completed high school, and completed technical school or university), income earned in the previous week (categorized by quartile), occupation (formal worker (government or private sector), independent worker, student, housewife, unemployed), marital status (single/divorced/widowed, married/cohabitating), health insurance (yes/no), migration directly to the community from a location outside of the Lima metropolitan area, overcrowding (as measured by the number of people living in the home), access to an improved water source, and access to improved sanitation. A forward stepwise approach was used to identify confounding variables and those variables having a corresponding p-value of less than 0.05 were retained in the final model. Data were collected and managed using REDCap electronic data capture tools [29] and analyzed using STATA, version 13.1 (StataCorp, College Station, TX). A significance level of 0.05 was used for all hypothesis testing.

## Results

### Study population

211 households were visited by field workers, with 193 households providing basic demographic information (age and gender of household members) and 145 households completing study procedures. Three households were excluded from the final sample because their inclusion would have resulted in oversampling of the cluster, yielding a final sample of 142 households for analysis.

Seventy-four percent of selected household respondents were successfully contacted and accepted to participate, with females being significantly more likely to participate as compared to males (85.3% vs. 55.8%; p < 0.001). Males were more likely to be unsuccessfully contacted (22.1% vs. 6.9%, p = 0.001) and were more likely to refuse participation (22.1% vs. 7.8%, p = 0.001) and as compared to females. Study participants and non-participants did not differ significantly by age (p = 0.46).

Fifty percent of the study population was female, and the mean age was 35.9 years (95% CI: 32.3, 39.6). The mean household size was 5.4 members (95% CI 4.8, 5.9), half of subjects had an elementary school education or less, and 59.2% were married or cohabitating. Household income ranged from 30 to 900 soles (about US$12-346) weekly, with a mean of 268 soles (about US$100). Subject sociodemographic characteristics are summarized in Table 1.

**Table 1:** Characteristics of 142 adults in a shantytown in Lima, Peru, 2012

**Characteristic**	**Total (N = 142)**	**Male (N = 43)**	**Female (N = 99)**	
	**%**	**%**	**%**	**p-value**
**Age (years)**				0.23
18-25	25.9	25.7	26.0	
26-35	32.4	37.7	27.2	
36-45	22.5	14.1	30.8	
46+	19.3	22.5	16.0	
**Marital status**				0.10
Single	36.8	46.3	27.4	
Married	18.8	22.1	15.5	
Separated	3.0	2.3	3.6	
Widowed	1.0	0.0	2.1	
Cohabitating	40.4	29.3	51.5	
**Highest level of education attained**				0.81
Less than primary	14.0	13.7	14.3	
Primary	36.4	33.9	38.8	
Secondary	43.1	47.1	39.2	
Technical institute	5.8	5.3	6.3	
University (non-technical)	0.7	0.0	1.4	
**Weekly household income quartile (soles)**				0.69
<125	22.3	20.6	24.0	
125-200	31.8	26.9	36.7	
201-300	22.7	24.7	20.8	
>300	23.1	27.8	18.5	
**Mean household size (95% CI)**	5.4 (4.8-5.9)	5.5 (4.7-6.3)	5.3 (4.7-5.9)	0.05
**Health insurance**^**†**^	30.4	24.4	36.4	0.16
**Migration from outside Lima-Callao**	42.7	49.0	36.4	0.21

^**†**^Types of health insurance reported included Essalud, Seguro Integral de Salud (SIS), police or armed forces insurance, and private health insurance.

Although nearly all respondents (98.6%) reported receiving medical care the last time they (or a child under age 13) needed it, improved access to medical care was the most frequently cited change respondents desired with regard to the health of adults living in their community (cited by 48.4% of respondents). Environmental improvements (paved roads, sidewalks, and the creation of green space) and improvements in health care quality were the next most frequently desired changes. The NCDs or NCD-related risk factors most commonly cited as important adult health issues in the community were poor nutrition, diabetes, cancer, and alcohol abuse.

### Prevalence of noncommunicable disease and risk factors

The most prevalent self-reported NCD diagnoses were depression (11.8%) and chronic respiratory disease (8.4%; Table 2). The prevalence of cancer, arthritis, myocardial infarction, and diabetes were all less than 5% (Table 2). Overweight/obesity and abdominal obesity were highly prevalent, as 52.6% of participants were overweight or obese and approximately 35% of participants had abdominal obesity (Table 2). Hypertension was less common, with a 15% prevalence (Table 2). Of participants who met the definition for hypertension, only 32.8% were aware of their condition. With regard to NCD behavioral risk factors, 21.1% of the population reported current smoking, but less than 1% reported doing so daily. Although only 39.3% of the population reported alcohol consumption within the previous month, about half (51.0%) of alcohol consumers binge drank on at least one occasion during that time period. Fruit and vegetable consumption was low overall, with subjects consuming an average of only one portion of fruit (mean 1.37, 95% CI 1.03, 1.70) and one portion of vegetables (mean 0.99, 95% CI 0.79, 1.20 daily). The large majority (91.6%) did not consume the recommended 5 portions of fruits and vegetables daily.

**Table 2:** Noncommunicable disease diagnoses and risk factors among adults in a shantytown in Lima, Peru, 2012

**Characteristic**	**Total (N = 142)**		**Male (N = 43)**		**Female (N = 99)**		**p-value**
	**%**	**(95% CI)**	**%**	**(95% CI)**	**%**	**(95% CI)**	
**Nutritional status ***(based on BMI)*							0.03
Normal weight	47.4	(37.1, 57.6)	60.0	(43.6, 76.4)	34.8	(23.1, 46.5)	
Overweight	39.5	(30.1, 48.9)	32.8	(16.6, 49.0)	46.2	(36.4, 56.1)	
Obese	13.1	(8.2, 18.1)	7.2	(0.0, 14.9)	19.0	(11.8, 26.2)	
**Abdominal obesity ***(WHO definition)*	31.9	(25.0, 38.9)	9.5	(1.0, 18.1)	54.3	(40.0, 68.5)	<0.001
**Abdominal obesity ***(Peruvian data definition)*	35.8	(28.1, 43.5)	16.4	(5.3, 27.5)	55.2	(40.9, 69.4)	0.001
**Hypertension**	14.9	(6.6, 23.3)	19.4	(6.0, 32.8)	10.5	(4.0, 17.0)	0.10
**Diabetes**	2.7	(0.0, 5.0)	1.5	(0.0, 4.6)	3.8	(0.0, 7.8)	0.42
**Depression**	11.8	(4.3, 19.4)	6.9	(0.0, 16.4)	16.8	(6.6, 26.9)	0.17
**Chronic respiratory disease**	8.4	(1.6, 15.1)	9.3	(0.0, 20.1)	7.4	(1.6, 13.3)	0.71
**Arthritis**	3.6	(0.0, 7.3)	1.9	(0.0, 5.9)	5.2	(0.0, 11.8)	0.40
**Cancer**	0.5	(0.0, 1.6)	0.0	(NA)	1.0	(0.0, 3.1)	0.32
**Myocardial infarction**	1.9	(0.0, 5.0)	3.1	(0.0, 9.2)	0.7	(0.0, 2.1)	0.26
**Current smoking**	21.1	(13.5, 28.6)	34.3	(18.7, 49.9)	7.9	(1.9, 13.9)	0.002
**Binge drinking in the past month**	19.4	(12.0, 26.8)	34.7	(20.7, 48.6)	4.1	(0.0, 8.3)	<0.001
**Inadequate fruit and vegetable consumption**	91.6	(86.2, 96.9)	89.7	(79.7, 99.8)	93.4	(88.8, 98.0)	0.47

### The effects of age and sex

**Figure 1:**
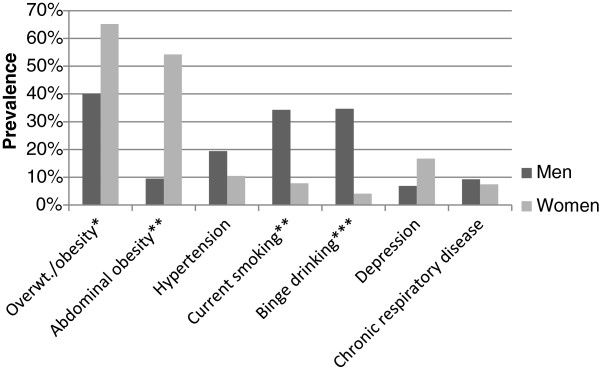
Gender-specific prevalences of selected noncommunicable disease diagnoses and risk factors in a Peruvian shantytown, 2012. Results are shown for univariate logistic regression models. Significance codes: p< 0.001 ‘***’, p< 0.01 ‘**’, p< 0.05 ‘*’, p> 0.05 ‘’.

**Figure 2:**
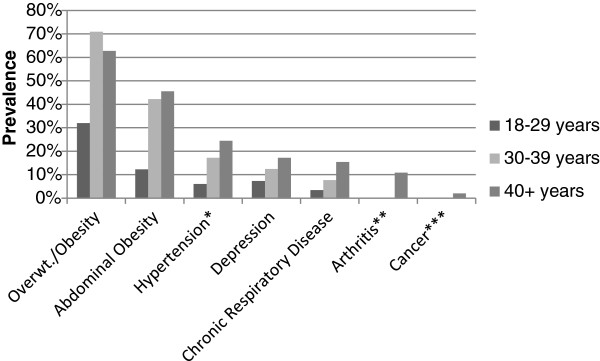
Age-specific prevalences of selected noncommunicable disease conditions and risk factors in a Peruvian shantytown, 2012. Results are shown for univariate logistic regression models, with significant p-values for the trend in continuous age denoted. Significance codes: p< 0.001 ‘***’, p< 0.01 ‘**’, p< 0.05 ‘*’, p> 0.05 ‘’.

We sought to identify the age and gender differences in the prevalence of noncommunicable disease diagnoses and risk factors in the study population (Figure 1, Figure 2). In univariate analysis, women were significantly more likely than men to be overweight or obese (65.2 vs. 40.0%, p = 0.02), and to have abdominal obesity (54.3 vs. 9.5% per WHO cutoffs, p = 0.002; 55.2 vs. 16.4% per Peruvian cutoffs, p = 0.001). In models adjusted for age and other confounding variables, the prevalence of overweight and obesity did not differ significantly by gender, however, females were six to thirteen times more likely to have abdominal obesity, depending on which definition of abdominal obesity was used (OR using WHO definition = 13.73, 95% CI = (2.71, 64.75), p = 0.002; OR using Peruvian data definition = 6.38, 95% CI = 1.71, 23.82, p = 0.007; Table 3). In univariate analysis, men were more likely to report current smoking (34.3 vs. 7.9%, p = 0.002) or binge drinking (34.7 vs. 4.1%, p < 0.001). These differences correspond to a five-fold increased likelihood of current smoking (OR = 6.21, 95% CI = 1.83, 21.10, p = 0.005) and thirteen-fold increased risk of binge drinking (OR = 14.02, 95% CI = 3.66, 53.71, p < 0.001) as compared to females in adjusted models (Table 3). Men and women did not differ significantly with respect to the prevalence of other chronic disease conditions or risk factors although the association between gender and cancer risk could not be evaluated due to small numbers of cases. In models adjusted for gender and other confounding variables, increasing age was associated with an increased risk of abdominal obesity (WHO definition; Odds Ratio (OR) = 1.04, 95% CI = 1.01, 1.07, p = 0.01), hypertension (OR = 1.06, 95% CI = 1.02, 1.10, p = 0.006), arthritis (OR = 1.08, 95% CI = 1.04, 1.12, p = 0.001 per year), and cancer (OR = 1.13, 95% CI = 1.07, 1.20, p = 0.001). There were no other significant differences in the prevalence of NCD or related risk factors by age (Tables 3 and 4).

**Table 3:** Associations of sex and age with noncommunicable disease risk factors in a Peruvian shantytown, 2012

	**Overweight/obesity**^**a**^	**Obesity**^**b**^	**Abdominal obesity**^**b **^***(WHO definition)***	**Abdominal obesity *(Peruvian data definition)***	**Hypertension**^**c**^	**Current smoking**	**Binge drinking**^**d**^	**Inadequate fruit and vegetable consumption**^**d**^
	**Multivariable OR**	**Multivariable OR**	**Multivariable OR**	**Multivariable OR**	**Multivariable OR**	**Multivariable OR**	**Multivariable OR**	**Multivariable OR**
	**(95% CI)**	**(95% CI)**	**(95% CI)**	**(95% CI)**	**(95% CI)**	**(95% CI)**	**(95% CI)**	**(95% CI)**
Female	2.15	2.51	13.03**	7.41**	0.44	0.16**	0.07***	1.76
Gender	(0.80, 5.78)	(0.63, 10.05)	(2.76, 61.50)	(2.10, 26.20)	(0.18, 1.12)	(0.05, 0.55)	(0.02, 0.27)	(0.42, 7.29)
Age	1.01	1.00	1.04*	1.04*	1.06**	0.98	0.97	1.01
(Years)	(0.98, 1.05)	(0.97, 1.04)	(1.00, 1.07)	(1.01, 1.08)	(1.02, 1.10)	(0.96, 1.01)	(0.93, 1.01)	(0.96, 1.06)

All models of associations of gender and risk factors are adjusted for continuous age.

All models of associations of age and risk factors are adjusted for gender.

^a^Model additionally adjusted for marital status, migration, and health insurance.

^b^Model additionally adjusted for marital status.

^c^Model additionally adjusted for household crowding.

^d^Model additionally adjusted for improved sanitation.

Significance codes: p < 0.001 ‘***’, p < 0.01 ‘**’, p < 0.05 ‘*’, p > 0.05 ‘’.

**Table 4:** Associations of sex and age with noncommunicable disease conditions in a Peruvian shantytown, 2012

	**Depression**	**Chronic respiratory disease**	**Arthritis**	**Cancer**	**Myocardial infarction**	**Diabetes**^**a**^
	**Multivariable OR**	**Multivariable OR**	**Multivariable OR**	**Multivariable OR**	**Multivariable OR**	**Multivariable OR**
	**(95% CI)**	**(95% CI)**	**(95% CI)**	**(95% CI)**	**(95% CI)**	**(95% CI)**
Female	2.74	0.80	4.02	Could not be	0.22	2.58
Gender	(0.59, 12.74)	(0.21, 3.04)	(0.16, 100.26)	calculated	(0.01, 4.45)	(0.18, 37.20)
Age	1.00	1.01	1.07***	1.13***	0.98	1.06
(Years)	(0.97, 1.03)	(0.98, 1.04)	(1.03, 1.11)	(1.07, 1.20)	(0.95, 1.01)	(0.99, 1.14)

All models of associations of gender and disease conditions are adjusted for continuous age.

All models of associations of age and disease conditions are adjusted for gender.

^a^Model additionally adjusted for household crowding.

Significance codes: p < 0.001 ‘***’, p < 0.01 ‘**’, p < 0.05 ‘*’, p > 0.05‘.

## Discussion

In the context of a community health needs evaluation, we investigated the prevalence and age and gender distribution of noncommunicable disease in a peri-urban shantytown in Peru. Overall, there was a low prevalence of self-reported, diagnosed noncommunicable disease, although the prevalences of depression (12%) and chronic respiratory disease in the population (8%) signal that these conditions may contribute significantly to the NCD burden. Perhaps most alarming was the high frequency of excess weight, particularly among women; our findings of a 53% prevalence of overweight/obesity overall and 54% prevalence of abdominal obesity in women indicate a major risk for development of a noncommunicable disease. With regard to other risk factors for NCD, inadequate fruit and vegetable consumption by the majority (92%) of participants and a 15% prevalence of hypertension—the majority undiagnosed—in this population demonstrate the importance of these risk factors as potential contributors to NCD risk in this community.

Our estimates of NCD morbidity are generally similar to those of prior studies conducted in informal settlements in Peru [12,15,16]. However, our population prevalence of depression was substantially lower than the prevalence of probable mental illness estimated in the PERU-MIGRANT shantytown cohort (12 vs. 33-38%) [15]. This difference could be explained by our definition of depression as having received a diagnosis of depression from a healthcare provider, which likely underestimated the prevalence of depression in our population. The difference also may be due to the fact that in the PERU-MIGRANT study, current rather than lifetime prevalence was measured; recall bias may have resulted in underreporting of lifetime depression diagnoses. The measure employed in the PERU-MIGRANT study may additionally have resulted in an overestimate of population depression prevalence due to its inability to distinguish between depression and anxiety [15]. Our estimate of lifetime depression prevalence is, however, similar to that estimated for the adult population of metropolitan Lima (18%) [30]. As compared to mental health research conducted in slum populations outside of Peru, the prevalence of depression in the present study is less than half of that measured in older adults [31] and women [32] living in slums in India, and is lower than the prevalence of self-rated poor or fair mental well-being measured in two Bangladeshi slum populations [33,34]. These differences are likely attributable in part to the fact that in other studies the definition of depression or poor mental health did not require having received a physician diagnosis.

The only other study to our knowledge to report on the prevalence of chronic respiratory disease in a adult slum population, conducted in India, found similar prevalences of asthma symptoms (10%) and chronic bronchitis (8.5%) [35]. In this population, asthma symptomology, but not chronic bronchitis, was associated with female gender and increasing age. It is possible that the heterogeneity of conditions captured by our chronic respiratory disease measure obscured condition-specific gender and age differences that may have been present in our study population.

The prevalence of arthritis measured in the present study is slightly lower than the prevalence of osteoarthritis of the knees found in a Bangladeshi adult urban slum population [36], which may be attributable to differences in the working conditions and physical demands of occupations, particularly for men, in these populations. Diabetes prevalences estimated in slum populations in Kenya [37,38] and India [39] are similar to that of the present study while they are higher in Bangladesh (8%) [40] and in an elderly Indian population (18%) [41]. The paucity of cancer and myocardial infarction data from adult slum populations limits our ability to compare our findings regarding these outcomes.

Similarly to the prevalences of NCD conditions, the prevalences of risk factors for NCD estimated in this study are also comparable to those of prior studies in Peruvian informal settlements [12,13,17,18,42]. Our population prevalences of hypertension, overweight status, and obesity are slightly lower than those estimated for the PERU-MIGRANT shantytown cohort, however, the age-specific prevalences of these conditions in our population suggest that this difference is due to the slightly younger age of our study population. The prevalence of overweight and obesity estimated in this study was similar to that of other slum populations in Nigeria [43] and Kenya [37] but greater than that of a different Kenyan slum [44] and greater than Indian [39] and Bangladeshi [40] slum populations. These differences in the prevalence of overweight and obesity are likely due to a number of factors and may be related to the progress of the epidemiologic transition or characteristics specific to the particular slum setting. In contrast to the findings of the PERU-MIGRANT study [19], women did not have a significantly greater prevalence of obesity defined by BMI, however, the greater prevalences of abdominal obesity in women in our study suggest that this finding may have been due to the limitations of our sample size rather than a true difference in results. Like the finding of the current study, research in Kenyan [37,45] and Indian [39] slum populations found that women were more likely to have abdominal obesity while data from an Indian study provided evidence that abdominal obesity increased with age [39]. Age and gender associations with abdominal obesity were not assessed in the Nigerian or Bangladeshi studies and the association with age was not assessed in the Kenyan studies. The association we observed between female gender and abdominal obesity may be the result of gender norms affecting individual health-related behaviors such as physical activity. The association of abdominal obesity with age likely reflects the cumulative effects of unhealthy diet and insufficient physical activity over the lifespan which we did not investigate in the current study but may further investigate in future NCD studies in this population.

Comparing to slum populations outside of Peru, our population prevalence of hypertension was similar to that of populations in Kenya [45] and India [39], but was considerably less than the prevalence of 38% measured in a Nigerian slum population [43] and considerably greater than the approximately 2% prevalence measured in a Bangladeshi slum [40]. As in the present study, hypertension was associated with increasing age in previous studies conducted in Peru [13], India [39], Kenya [45] and Nigeria [43]; this association was not evaluated in the Bangladeshi population. This age-related trend with hypertension likely reflects the stiffening of blood vessels through ageing, although it could also reflect uncontrolled confounding by factors that are associated both with age and hypertension.

Like other research in informal settlements in Peru, we found a greater prevalence of binge drinking and smoking among men [17,18], which is similar to patterns of hazardous drinking and tobacco use in the adult population of the Lima metropolitan area [30] and which likely reflect differences in gender-based social expectations [18]. Similar gender differences in hazardous drinking and tobacco use have been noted in non-Peruvian slum populations [37,39,43,45].

Because this study was conducted in the context of a broader health needs assessment, we designed our study instruments with the goal of balancing three objectives: 1) to capture a large number of health indicators on a broad spectrum of topics to describe the community burden of disease, 2) to collect valid measures of NCD-related variables, and 3) to limit participant burden. Although we did obtain an overall picture of the burden of communicable and noncommunicable disease in this population, we were unable to collect a number of NCD and NCD risk-related variables or to use more time-consuming, higher validity measures for each variable. The self-reported nature of the NCD variables, for example, likely led to underestimates of the prevalence of these diseases. With regard to NCD-related risk behaviors, only one aspect of unhealthy diet—adequate fruit and vegetable consumption—was measured, and we did not measure this variable using a high validity measure such as a food frequency questionnaire. Future investigations of diet-related NCD risk factors could additionally collect information regarding salt intake and saturated fat and trans-fat consumption, two other important aspects of diet-related NCD risk [8]. Due to concerns about survey length, we also did not measure physical activity in our survey, and the inclusion of this risk factor will be important in future community surveys both as an important risk behavior related to NCD and to measure the impact of interventions and improvements in community infrastructure. The inclusion of biochemical measures would have allowed us to better measure certain NCDs and associated risk factors such as diabetes and high cholesterol and therefore should be included in future NCD studies in this community. Although our population estimates were weighted to account for the greater proportion of non-participation among males selected to participate in this study, the difficulty we encountered in contacting and in obtaining consent from potential male participants highlights the importance of coordinating the schedule of data collection with the availability of less accessible segments of the population and the potential need for participation incentives in order to reduce underrepresentation of men in the sampling of similar populations. Our relatively small sample size may have precluded the identification of other differences in NCD and risk factor prevalence by age and gender.

Despite these limitations, this study had a number of strengths. To our knowledge, this is the first population-based study to describe the burden of noncommunicable disease and related risk factors in a Peruvian shantytown. Aside from the randomized sampling and novel population of this study, other strengths include the objective measurement of BMI, abdominal obesity, and hypertension, and the use of a standardized questionnaire to assess NCD risk behaviors.

## Conclusions

Our study findings provide a panorama of the prevalence of noncommunicable disease and related risk factors in informal settlements in Peru, highlighting the alarming frequency of overweight status and obesity. This study also identified age and gender as determinants of a number of NCDs and risk behaviors, thereby lending support to observations that health interventions in informal settlements may be optimized by age- and gender-specific approaches [46]. Among men, the approximately 35% prevalences of binge drinking and tobacco use highlight these behaviors as potential foci of NCD prevention interventions. Effective interventions are needed to reduce NCD in these communities. Specifically, community-based interventions comprising primary prevention, detection, and treatment of noncommunicable disease such as the cardiovascular disease intervention package currently being tested in slum communities in Nairobi, Kenya [47] may offer a potential way forward. Future research on interventions to decrease NCD and NCD risk factor prevalence should also assess cost-effectiveness and sustainability in order to ensure future reductions in the NCD disease burden. Investment in research to identify low-cost interventions is crucial to avoid the far higher long-term cost of continued neglect of the urban poor in the developing world.

## Abbreviations

BMI: Body mass index; NCD: Noncommunicable disease; WHO: World Health Organization.

## Competing interests

The authors declare that they have no competing interests. Dr. Silvia Montano is an employee of the U.S. Government. This work was prepared as part of her official duties. Title 17 U.S.C. §105 provides that 'Copyright protection under this title is not available for any work of the United States Government'. Title 17 U.S.C. §101 defines a U.S. Government work as a work prepared by a military service member or employee of the U.S. Government as part of that person's official duties. The views expressed in this manuscript are those of the authors and do not necessarily reflect the official policy of position of the Department of the Navy, Department of Defense, nor the U.S. government.

## Authors’ contributions

All authors participated in the design of the study. KH conceived of the study, supervised and participated in data collection, performed the statistical analysis and drafted the manuscript. SM and JA assisted in the coordination of the data collection. SM, SH, JA, and JZ provided critical feedback on drafts. All authors read and approved the final manuscript.

## Pre-publication history

The pre-publication history for this paper can be accessed here:

http://www.biomedcentral.com/1472-698X/14/19/prepub

## Supplementary Material

Additional file 1 Noncommunicable diseases and risk factors survey. Click here for file
