# Supplementary material for: A community-based cluster randomized survey of noncommunicable disease and risk factors in a peri-urban shantytown in Lima, Peru
Source: BMC Int Health Hum Rights. 2014 May 21;14:19. doi: 10.1186/1472-698X-14-19 (PMC4040114; doi:10.1186/1472-698X-14-19)
Supplement: Additional file 1 — Noncommunicable diseases and risk factors survey. [file 1472-698X-14-19-S1.doc]

**A Quantitative Health Needs Assessment in Lomas de Zapallal, Lima, Peru**

**Household Sampling and Respondent Selection**

| Location and Date | | Response |
| --- | --- | --- |
| S1 | Lot Number | └─┴─┴─┘ |
| S2 | Block Number | └─┴─┴─┘ |
| S3 | Interview Date(e.g. 01-JAN-2012) | **2 0 1 2**  └─┴─┘ └─┴─┴─┘ └─┴─┴─┴─┘ dd month year |
| S4 | Interviewer Code | └─┴─┴─┴─┘ |

**Roster of Adults (Age 18 and Older) Living in the Household**

| Any information you provide is strictly confidential and will not be used for any purpose other than scientific research. It is not necessary to provide us with the names of the people living in your household. I would only like the age and sex of each of the adults (age 18 and older) who currently live here.* Please include people who may presently be in an institution due to their health (hospital, nursing home etc.) for a short or long period. **Don’t forget to include yourself in the appropriate order.** | | | | | | |
| --- | --- | --- | --- | --- | --- | --- |
| A  Sex  (M=male  F=female) | B  Age  (years) | C  Eligible person?*  (If is elegible, mark with “X”) | D  Person No. | E  Selected Interviewee  (Mark with “X”) | F  Availability of Interviewee | G  Visit Date  (in case of a 2^nd^ or 3^rd^ visit) |
|  |  |  |  |  |  |  |
|  |  |  |  |  |  |  |
|  |  |  |  |  |  |  |
|  |  |  |  |  |  |  |
|  |  |  |  |  |  |  |
|  |  |  |  |  |  |  |
|  |  |  |  |  |  |  |
|  |  |  |  |  |  |  |
|  |  |  |  |  |  |  |
|  |  |  |  |  |  |  |
| To confirm, please repeat the list. | | | | | | |

IF INCORRECT, CORRECT LISTING.

*An eligible person meets the following criteria: 1) Is 18 years of age or older 2) Usually sleeps and shares meals in the household, uses the household address as his permanent address, **or** spends at least 6 months of the year living in the household and 3) does not have any physical or mental disability that would prevent completion of the study procedures.

| Now, I am going to randomly select an person from the list to interview. (This will take a minute.) |
| --- |

IF NO ONE AGE 18 OR OLDER IS IN HOUSEHOLD ROSTER, THEN THERE IS NO ELIGIBLE RESPONDENT AND YOU MAY CONTINUE TO THE NEXT HOUSEHOLD. IF THERE IS AN ELIGIBLE RESPONDENT, INVITE RESPONDENT TO PARTICIPATE IN STUDY, COMPLETE INFORMED CONSENT AND SURVEY.

**A Quantitative Health Needs Assessment in Lomas de Zapallal, Lima, Peru**

**Participant Identification Information**

| Consent and Name | | Response | |
| --- | --- | --- | --- |
| **P1** | Consent has been read and obtained | Yes | 1 |
|  |  | No | 2 **IF NO, END** |
| P2 | Family Surname(s) |  | |
| **P3** | First Name(s) |  | |
| **P4** | Contact phone number |  | |
| **P5** | Date of Interview  (Ex. 01-JAN-2012) |  | |
| **P6** | Interviewer Code | **└─┴─┴─┘** | |

RECORD AND FILE IDENTIFICATION INFORMATION (QUS. P1-P4) SEPARATELY FROM THE COMPLETED QUESTIONNAIRE.

**A Quantitative Health Needs Assessment in Lomas de Zapallal, Lima, Peru**

**Participant Survey**

**SECTION 1: DEMOGRAPHICS**

**1.**  Sex (1) Male (2) Female |__|

**2. a.** What is your date of birth? |__|__| |__|__|__| 19|__|__|

DD MMM YY

**b.** CALCULATE THE AGE OF THE PARTICIPANT: |__|__| YEARS

**3.** What is your marital status? |__|

(1) single (never married) (2) married (3) separated (4) divorced

(5) widowed (6) cohabitating (8) other, specify:__________

**4.** What is the highest grade of education you have attained? |__|

(1) No formal Schooling (4) Superior- not university (Technical School)

(2) Primary school completed (5) Superior- university

(3) High school completed (6) Postgraduate degree

**5.** During the last 12 months, what has been your **main occupation**? |__|

(1) Government employee (5) Homemaker/caring for family

(2) Nongovernment employee (6) Retired

(3) Self-employed (7) Unemployed (can work)

(4) Student (8) Unemployed (cannot work)

**6.** How many people, including yourself and any minors, live in your household? |__|__|

**7.** During **the past week**, can you tell me what the average earnings of the household have been? ___________ soles/week (999) Refused *(Include income of men, women, and children, if applicable)*

**8.** Are you enrolled in any of the following: ESSALUD, Seguro Integral de Salud, or another type of health insurance? (1) Yes (0) No (9) Unknown |__|

**If Yes:** In what? |__|

(1) ESSALUD/Antes IPSS (2) Armed or Police Forces

(3) Seguro Integral de Salud (4) Private Health Insurance

**9. a.** How long have you been living in Lomas de Zapallal? |__|__| |__|__|

(99 year, 99 months = entire life; If entire life, go to Qu. 10) years months

**b.** Before moving to Lomas de Zapallal, where did you live? |__|

1. Lima (2) Outside of Lima, specify: _____________________

**SECTION 2: HOUSEHOLD CHARACTERISTICS AND ENVIRONMENTAL HEALTH**

**10. a. In the past 2 weeks,** has any member of your household had diarrhea? (1/0/9)|__|

**b. If Yes:** Who? How old is this person(s)? ______________________

*(Note the age of each person mentioned.)*

**11. a.** What is the main source of cooking/drinking water of your household? |__|

**Piped water Covered well (or with pump) Uncovered well**

(1) inside the house (4) Inside the house (6) Inside the house

(2) outside the house (5) Public/communal well (7) Public/communal well

(3) public standpipe

(8) Tanker truck/water carrier

(9) Other, specify:____________________

**b. If piped water:**

1) Is the water supply available all day? (1/0/9) |__|

2) In the past 2 weeks, has the water supply been interrupted for a day or more? (1/0/9) |__|

**12. a.** In your household, do you usually drink water directly from the pipe, well, or other source? (1/0/9) |__|

**b. If No: 1)** How do you treat the water that you usually use for drinking? |__|

(1) Boiling (2) Use bleach/chlorine (3) Use a water filter

(4) Solar disinfection (5) Let it stand (6) Use bottled water

(8) Other, specify: _____________

**13.** What kind of toilet is your household using most? |__|

(1) toilet connected to the sewage system (2) toilet connected to a well/septic tank (3) improved latrine (4) unimproved latrine (5) No sanitation (in a field) (8) Other, specify: ______________________________

**SECTION 3: HEALTH STATE DESCRIPTIONS**

**14.** In general, would you say your health is: |__|

(1) Very good (2) Good (3) Fair (4) Poor (5) Very poor (9) Unknown/Refused

**15.** Overall in the last 30 days, how much difficulty did you have with self-care, such as washing and dressing yourself?|__|

1. None (1) Mild (2) Moderate (3) Severe (4) Extreme/cannot do

**16.** Overall in the last 30 days, how much bodily ache or pain did you have? |__|

1. None (1) Mild (2) Moderate (3) Severe (4) Extreme

**17. a.** Do you wear glasses or contact lenses? (1) Yes (0) No (9) Refused|__|

**b.** Overall in the last 30 days, how much difficulty did you have with seeing and recognizing a person across the road (i.e. from a distance of 20 meters)?|__|

*(Please answer the following questions taking into account your glasses or contact lenses)*

1. None (1) Mild (2) Moderate (3) Severe (4) Extreme/Cannot do

**SECTION 4: BEHAVIORAL MEASURES**

**TOBACCO USE**

**18.** Do you currently smoke any **tobacco products**, such as cigarettes, cigars or pipes? (1/0/9) |__| (If No, go to question 32)

**19. a.** Do you currently smoke tobacco products **daily**? (1/0/9) |__|

(If No, go to question 32)

**b.** How old were you when you **first started** smoking daily? Age (years) |__|__| (88) Don´t know

**c.** On average, **how many** of the following do you smoke each day? (88) Don´t know

Manufactured cigarettes |__|__|

Hand-rolled cigarettes |__|__|

Pipes full of tobacco |__|__|

Cigars, cheroots, cigarillos |__|__|

Other |__|__| If other, specify: _____________

**DRINKING**

**20. a.** Have you **ever** consumed an alcoholic beverage? (1/0/9) |__|

(If No, go to Qu. 34)

**b.** Have you consumed an alcoholic drink within the **past 12 months**? (1/0/9) |__|

(If No, go to Qu. 34)

**c.** During the past 12 months, **how frequently** have you had at least one alcoholic drink? |__|

1. Daily (2) 5-6 days/week (3) 1-4 days/week (4) Less than once a month

**21. a.** Have you consumed an alcoholic drink within the **past 30 days**? (1/0/9) |__|

(If No, go to Qu. 34)

**b.** During the past 30 days, on how many **occasions** did you have at least one alcoholic drink? |__|__| (99) Don´t know

**c.** During the past 30 days, when you drank alcohol, **on average**, how many **standard** **alcoholic** **drinks** did you have during one drinking occasion? |__|__| (99) Don´t know

**d.** During the past 30 days, what was the **largest number** of standard alcoholic drinks you had on a single occasion, counting all types of alcoholic drinks together? |__|__| (99) Don´t know

**e.** During the past 30 days, how many times did you have

for **men**: **five or more**

for **women**: **four or more**

standard alcoholic drinks in a single drinking occasion? |__|__| (99) Don´t know

**DIET**

**22. a.** In a typical week, on how many days do you **eat fruit**? |__|

(9) Don´t know/Refused (If zero days, go to Qu. 35)

**b.** How many **servings** of fruit do you eat on **one** of those days? |__|__|

(9) Don´t know/Refused

(*A serving of fruit is 1 medium piece of fruit, ½ cup chopped, cooked, canned fruit or fruit juice)*

**23. a.** In a typical week, on how many days do you **eat vegetables**? |__|

(9) Don´t know/Refused (If zero days, go to Qu. 36)

**b.** How many **servings** of vegetables do you eat on **one** of those days? |__|__|

(9) Don´t know/Refused

(*A serving of vegetables is 1 cup raw green leafy vegetables, ½ cup other vegetables, or ½ cup vegetable juice)*

**SECTION 5: DIAGNOSIS AND TREATMENT OF CHRONIC DISEASE**

**24.** Have you been told by a doctor or other health worker that you have (had) any of the following: (write age at 1^st^ diagnosis)

**a.** Heart Attack (1/0/9) |__| Age at dx: __ __

**b.** Cancer (1/0/9) |__| Age at dx: __ __ Type: _______________

**c.** Arthritis (1/0/9) |__| Age at dx: ___ ___

**d.** Chronic respiratory |__| Age at dx: ___ ___ Specify condition: ____________

*(Ex. asthma, chronic bronchitis, COPD)*

**e.** Depression (1/0/9) |__| Age at dx: ___ ___

**During the last 12 months,** have you had:

**f.** A cough with phlegm that lasted for 3 weeks or more? (1/0/9) |__|

**g.** Blood in your phlegm or have you coughed blood? (1/0/9) |__|

**h.** A tuberculosis (TB) test? I mean, has a doctor examined your sputum (taken a sample of the substance spit out from a deep cough and sent it to a laboratory for analysis) or made an x-ray of your chest)? (1/0/9) |__|

**25. a.** Have you ever had your blood pressure measured by a doctor or other health worker? (1/0/9) |__| (If No, go to Qu. 26)

**b.** Have you ever been told by a doctor or other health worker that you have raised blood pressure or hypertension? (1/0/9) |__| (If No, go to Qu. 26)

**c.** If yes: have you been told in the past 12 months? (1/0/9) |__|

**26. a.** Have you ever had your blood sugar measured by a doctor or other health worker? (1/0/9) |__|

**b.** Have you ever been told by a doctor or other health worker that you have raised blood sugar or diabetes? (1/0/9) |__| (If No, go to Qu. 27)

**c.** Have you been told in the past 12 months? (1/0/9) |__|

**27. During the past 12 months:**

**a.** Have **you or** **anyone in your household been the victim of a violent crime,** such as assault or rape? (1/0/9) |__|

**b.** Were you injured in a car accident? (1/0/9) |__|

**c.** Suffered an injury that limited your daily activities due to a fall, burn, poisoning, submersion in water, or firearm, sharp object, or violent act of another person? (1/0/9) |__|

**ORAL HEALTH**

| Now I would like to ask you some questions about the condition of your mouth and teeth. |
| --- |

**28.** During **the last 12 months,** did you have any problems with your mouth and/or teeth? (1/0/9) |__| If Yes, specify: ____________________ (If No, skip to the next section)

**29.** During **the last 12 months,** did you receive any medical care or treatment from a dentist or oral health specialist for this problem with your mouth and/or teeth? (1/0/9) |__|

**SECTION 6: HEALTH SYSTEM RESPONSIVENESS**

**30.** When was the last time that either you as an adult, or a child of yours aged 12 years or less, needed health care? |__|

(1) In the last 30 days

(2) Between 1 month and less than 1 year ago

(3) Between 1 year and less than 2 years ago

(4) Between 2 years and less than 3 years ago (If 7, go to Qu. 38)

(5) Between 3 years and less than 5 years ago

(6) More than 5 years ago

(7) Never needed

**31.** Was the last need for health care for yourself or for your child? |__|

1. Yourself (2) Your child

**(Interviewer: Use “you” or “your child” according to the response.)**

**32.** Which reason **best** describes why you (your child) last needed health care? |__|

(1) Dental care (2) Arthritis (3) Gastrointestinal infection (4) Respiratory Infection (5) Minor surgery

(5) Other, specify:_________

**33.** The last time you (your child) needed health care, did you get health care?

(1/0/9) |__| (If Yes, go to Qu. 37)

**34.** Which reasons **best** explain why you (your child) **did not** get health care?

Could not afford the cost of the visit (1) Yes (0) No |__|

No identification (1) Yes (0) No |__|

No transport (1) Yes (0) No |__|

Could not afford the cost of transport (1) Yes (0) No |__|

The health care provider´s drugs or equipment are inadequate (1) Yes (0) No |__|

The health care provider´s skills are inadequate (1) Yes (0) No |__|

You were previously badly treated (1) Yes (0) No |__|

Could not take time off work or had other commitments (1) Yes (0) No |__|

You did not know where to go (1) Yes (0) No |__|

You thought you were not sick enough (1) Yes (0) No |__|

You tried but were denied health care (1) Yes (0) No |__|

Other, Specify:_____________ (1) Yes (0) No |__|

**35.** When you last needed health care, where did you get care? |__|

(1) At a health care provider, excluding overnight stay

(2) At a hospital where you stayed overnight

(3) At home (8) Other, Specify: ______________

**36.** In general would you say you are **very** satisfied, **fairly** satisfied, neither satisfied nor dissatisfied, **fairly** dissatisfied, or **very** dissatisfied with the way health care runs in your community? |__|

(1) very satisfied

(2) fairly satisfied

(3) neither satisfied nor dissatisfied

(4) fairly dissatisfied

(5) very dissatisfied

**37. a.** During the past year, did you **provide help** to a relative or friend (adult or child) because this person has a long-term physical or mental disability or is getting old and weak? (1/0/9) |__|

**b. If Yes,** Does this person live in your household or a different one?

(1) Yes, for a person living in the same household

(2) Yes, for a person living in a different household

Specify the reason for which the person needs help:

_________________________________________

*(Specify the type of physical or mental disability or write “old” if is getting old and weak)*

**COMMUNITY HEALTH PRIORITIES**

**38.** What are the three most important health problems for **adults** living in Lomas de Zapallal?

1.____________________________________________

2. ____________________________________________

3. ____________________________________________

**39.** What are the three most important health problems for **children** living in Lomas de Zapallal?

1.____________________________________________

2. ____________________________________________

3. ____________________________________________

**40.** What changes would you like to see with respect to the health of this community?

__________________________________________________

**41.** Who do you think should be responsible for making these changes?

__________________________________________________

**MEDICATIONS AND TRADITIONAL HOME REMEDIES**

**42**. Have you taken any medications over the past 2 weeks? |___|

(0) No, takes nothing / has taken nothing over the past 2 weeks

(1) Yes, they are documented here

(9) Unknown / Refused

| **If Yes:** Can you please collect all of these for me? Please bring any that you have taken including those you have taken daily and those taken only once or a few times in the past two weeks. Include vitamins, pills for headache, pain, etc. |
| --- |

**43**. Are any pharmacy medications taken but unavailable for transcription (0 = none)? |___|

Allopathic Medications and Supplements from a Doctor or Pharmacy

| Medication Name | Strength (mg, iu, etc) | Number taken in last 2 weeks |
| --- | --- | --- |
|  |  |  |
|  |  |  |
|  |  |  |
|  |  |  |
|  |  |  |

1. In the last 2 weeks, have you taken any traditional (alternative medicine) supplements or therapies including herbs, ayurvedic, homeopathic or traditional home remedies? |___|

(0) No (1) Yes (9) Unknown / Refused

If yes, please list and provide reason that you take them:

**Therapy** **Reason taken**

__­­­­__­­­­­­­­­­­­­­­­­­­­­­­­­­­­­­­­­­­­___________________________ ___________________

__­­­­__­­­­­­­­­­­­­­­­­­­­­­­­­­­­­­­­­­­­___________________________ ___________________

__­­­­__­­­­­­­­­­­­­­­­­­­­­­­­­­­­­­­­­­­­­­___________________________ ___________________

**PHYSICAL MEASUREMENTS**

**46.** Height in centimeters |__|__|__|.|__| cm

**47.** Weight in kilograms |__|__|.|__| kg

**For women:** Are you pregnant? (1) Yes (0) No |__|

*(If yes, skip weight circumference qu.)*

**48.** Waist circumference in centimeters |__|__|__|.|__| cm

**49.** Blood Pressure

First Reading |___|___|___| |___|___|___|

Systolic Diastolic

Second Reading (after 30’’) |___|___|___| |___|___|___|

Systolic Diastolic

**ASSESSMENT OF ADULT MORTALITY**

**Section A1 : Sibling Survivorship**

| 1. How many children did your mother give birth to, including you? Number of births to biological mother: |  |
| --- | --- |
| 2. How many births did your mother have before you were born? Number of preceding births: |  |
| 3. How many births did your mother have after you were born? Number of suceeding births: |  |

**INTERVIEWER: INCLUDE ALL SIBLINGS (E.G. STEP SIBLINGS, BORN TO THE SAME MOTHER). IF UNKNOWN, WRITE 99.**

| Now I would like you to list for me details of all your siblings from oldest to youngest (including yourself). |
| --- |

COMPLETE COLUMNS FOR ALL SIBLINGS. IF PARTICIPANT DOES NOT HAVE SIBLINGS, SKIP TO THE NEXT SECTION.

| If 2 of more births (1 or more siblings), continue here, starting with the oldest child: | Sibling 1 | | Sibling 2 | | Sibling 3 | | Sibling 4 | | Sibling 5 | | Sibling 6 | | Sibling 7 | | Sibling 8 | |
| --- | --- | --- | --- | --- | --- | --- | --- | --- | --- | --- | --- | --- | --- | --- | --- | --- |
| Name |  | |  | |  | |  | |  | |  | |  | |  | |
| Sex (M=male, F=female) | M | F | M | F | M | F | M | F | M | F | M | F | M | F | M | F |
| Month/Year of birth (i.e. Feb-02) |  | |  | |  | |  | |  | |  | |  | |  | |
| What is the age difference (in years) between you and [NAME]? |  | |  | |  | |  | |  | |  | |  | |  | |
| Is [NAME] still alive? (0/1)  *(If Yes, skip to the next sibling)* |  | |  | |  | |  | |  | |  | |  | |  | |
| **FOR DECEASED SIBLINGS:**  How old was [NAME] when s/he died? |  | |  | |  | |  | |  | |  | |  | |  | |
| How many years ago did [NAME] die? |  | |  | |  | |  | |  | |  | |  | |  | |

**Section A2: Verbal Autopsy**

FOR EACH SIBLING DEATH RECORDED IN SECTION A1, ANSWER THE FOLLOWING QUESTIONS. COMPLETE COLUMNS FOR ALL SIBLINGS.

|  | Sibling 1 | Sibling 2 | Sibling 3 | Sibling 4 | Sibling 5 | Sibling 6 | Sibling 7 | Sibling 8 |
| --- | --- | --- | --- | --- | --- | --- | --- | --- |
| 1. a. Was the deceased a woman aged 15-49 when she died? (1/0/9)  *(If No, skip to Qu. 2)* |  |  |  |  |  |  |  |  |
| b. Was she pregnant when she died? |  |  |  |  |  |  |  |  |
| 1. Did she die during childbirth? |  |  |  |  |  |  |  |  |
| 1. Did she die within 2 months after the end of pregnancy or childbirth? |  |  |  |  |  |  |  |  |
| 2. a. Was the death associated with injury? (1/0/9)  *(If No, skip to the Qu. 3)* |  |  |  |  |  |  |  |  |
| b. Was it due to:   1. Accident (2) Suicide (3) Murder (4) War 2. Natural disaster (6) Other, specify:___________________ |  |  |  |  |  |  |  |  |
| c. Provide details of events that led to the injury. What was the mechanism or cause of injury?  (1) Motor vehicle (6) Gunshot, firearm related  (2) Pedestrian-vehicle crash (7) Stabbing  (3) Motorcycle (8) Fire/burn  (4) Pedal cycle (9) Other, specify:______________  (5) Fall |  |  |  |  |  |  |  |  |
| d. Where did the injury occur?  (1) Home (2) School (3) Street/highway  (4) Other, specify:________ |  |  |  |  |  |  |  |  |
| 3. Did the deceased have chest pain lasting less than 24 hrs in the month preceding the death? |  |  |  |  |  |  |  |  |
| 4. a. Did the deceased experience paralysis of any part of the body in the month preceding death? |  |  |  |  |  |  |  |  |
| b. If Yes, was the paralysis accompanied or followed by sudden loss of consciousness? |  |  |  |  |  |  |  |  |
| 5 a. Did the deceased have a cough that lasted more than 3 weeks? |  |  |  |  |  |  |  |  |
| b. If Yes, was there blood in the sputum? |  |  |  |  |  |  |  |  |
| 6. Did s/he receive any medical treatment for tuberculosis? |  |  |  |  |  |  |  |  |
| 7. Did the deceased have diarrhea that lasted more than a month? |  |  |  |  |  |  |  |  |
| 8. Was there any rapid loss of weight? |  |  |  |  |  |  |  |  |
| 9. Were there any white patches in the mouth? |  |  |  |  |  |  |  |  |

**INSTRUCTIONS FOR THE INTERVIEWER:**

| IF THE PARTICIPANT IS MALE🡪 | END SURVEY |
| --- | --- |
| IF THE PARTICIPANT IS A WOMAN AGE 70 OR OLDER🡪 | END SURVEY |
| IF THE PARTICIPANT IS A WOMAN AGE 18-69 YEARS🡪 | CONTINUE TO THE NEXT SECTION |

***CERVICAL CANCER AND BREAST CANCER SCREENING (***Women age 18-69 only)

| Now I would like to ask you about some of the kinds of medical care that you have received. |
| --- |

**50. a.** When was the last time that you had a pelvic examination?

*(By pelvic examination, I mean when a doctor or nurse examined your vagina and uterus)*

(1) Within the last 3 years (2) 4-5 years ago (3) More than 5 years ago (8) Never had exam |__|

(IF MORE THAN 5 YEARS AGO OR NEVER, GO TO QU. 51)

**b.** The last time you had a pelvic examination, did you have a pap smear test? (1/0/9) |__|

*(By Pap smear test, I mean did a doctor or nurse use a swab or stick to wipe from the inside of your vagina, take a sample and send it to a laboratory?)*

**INSTRUCTIONS FOR INTERVIEWER: VERIFY THE WOMAN’S AGE.**

| IF 50-69 YEARS🡪 | COMPLETE THE NEXT QUESTION AND END SURVEY |
| --- | --- |
| IF 40-49 YEARS🡪 | COMPLETE THE NEXT QUESTION AND BIRTH HISTORY |
| IF 18-39 YEARS🡪 | SKIP THE NEXT QUESTION AND COMPLETE BIRTH HISTORY |

**51.** When was the last time you had a mammography, if ever? |__|

*(An x-ray of your breasts taken to detect breast cancer at an early stage.)*

(1) Within the last 3 years (2) 4-5 years ago (3) More than 5 years ago (8) Never had exam (9) Don´t know/Refused

***BIRTH HISTORY (Only Women 18-49 years)***

| Now, I would like to ask you about **all the births** you have had **during your life**. |
| --- |

*Have you ever given birth? (1/0/9) |__|* ***(IF NO, END SURVEY)***

|  | | Child 1 | | Child 2 | | Child 3 | | Child 4 | | Child 5 | | Child 6 | | Child 7 | | Child 8 | |
| --- | --- | --- | --- | --- | --- | --- | --- | --- | --- | --- | --- | --- | --- | --- | --- | --- | --- |
| **1.** Name | |  | |  | |  | |  | |  | |  | |  | |  | |
| **2.** Month/Year of birth (i.e. Feb-02) | |  | |  | |  | |  | |  | |  | |  | |  | |
| **3.** Sex *(Circle M=Male or F=Female)* | | M | F | M | F | M | F | M | F | M | F | M | F | M | F | M | F |
| **4.** Is he/she alive? | | Yes | No | Yes | No | Yes | No | Yes | No | Yes | No | Yes | No | Yes | No | Yes | No |
| **FOR LIVING CHILDREN** | |  | |  | |  | |  | |  | |  | |  | |  | |
| **For children older than 5 years and adults** | Current age in years | AA: | | AA: | | AA: | | AA: | | AA: | | AA: | | AA: | | AA: | |
| **For children between 2 months and 5 years of age** | Current age in months | MM: | | MM: | | MM: | | MM: | | MM: | | MM: | | MM: | | MM: | |
| **For infants below 2 months of age** | Current age in days | DD: | | DD: | | DD: | | DD: | | DD: | | DD: | | DD: | | DD: | |
| **Is the child currently living with you**? (0=No, 1=Yes) | |  | |  | |  | |  | |  | |  | |  | |  | |
| **FOR DECEASED CHILDREN** | |  | |  | |  | |  | |  | |  | |  | |  | |
| Month/year of death | |  | |  | |  | |  | |  | |  | |  | |  | |
| **For children older than 5 years and adults** | Age at death in years | DD: | | DD: | | DD: | | DD: | | DD: | | DD: | | DD: | | DD: | |
| **For children between 2 months and 5 years of age** | Age at death in months | MM: | | MM: | | MM: | | MM: | | MM: | | MM: | | MM: | | MM: | |
| **For infants below 2 months of age** | Age at death in days | AA: | | AA: | | AA: | | AA: | | AA: | | AA: | | AA: | | AA: | |
| Did s/he have fever? (0/1)  *(If No, skip to the question about convulsions).* | |  | |  | |  | |  | |  | |  | |  | |  | |
| - Was the fever continuous or on and off? | |  | |  | |  | |  | |  | |  | |  | |  | |
| - Was the fever associated with chills/shivering? (0/1) | |  | |  | |  | |  | |  | |  | |  | |  | |
| Did s/he have convulsions or fits? (0/1) | |  | |  | |  | |  | |  | |  | |  | |  | |
| Was the child unconscious for more than a day during the illness that led to death? (0/1) | |  | |  | |  | |  | |  | |  | |  | |  | |
| Did s/he have a stiff neck? (0/1) | |  | |  | |  | |  | |  | |  | |  | |  | |
| Did s/he have a cough? (0/1) *(Si No, skip to the question about breathing)* | |  | |  | |  | |  | |  | |  | |  | |  | |
| - If yes, was it (1) dry, (2) productive, (3) with blood, or (4) unknown? | |  | |  | |  | |  | |  | |  | |  | |  | |
| Did s/he have fast or difficult breathing? (0/1) | |  | |  | |  | |  | |  | |  | |  | |  | |
| Did s/he have diarrhea? (0/1) | |  | |  | |  | |  | |  | |  | |  | |  | |
| Was there visible blood in the stools? (0/1) | |  | |  | |  | |  | |  | |  | |  | |  | |
